# Functional differentiation of industrial hemp rhizosphere microbiome along environmental gradients

**DOI:** 10.3389/fpls.2025.1578662

**Published:** 2025-08-20

**Authors:** Jiayang Li, Hong Zhang, Tuhong Wang, Liya Peng, Kecheng Huang, Xiaoyu Tang, Wenting Li, Zhecheng Xu, Chao Li, Fengming Chen, Huajiao Qiu, Jianping Xu, Yi Cheng

**Affiliations:** ^1^ Hunan Provincial Key Laboratory of the Traditional Chinese Medicine Agricultural Biogenomics, Changsha Medical University, Changsha, China; ^2^ Institute of Bast Fiber Crops and Center of Southern Economic Crops, Chinese Academy of Agricultural Sciences, Changsha, Hunan, China; ^3^ Hunan Provincial Key Laboratory for Biology and Control of Plant Diseases and Insect Pests, Hunan Agricultural University, Changsha, Hunan, China; ^4^ Shenzhen Noposion Crop Science Co., Ltd., Shenzhen, Guangdong, China; ^5^ Shenzhen Inspection and Testing Center of Quality and Safety of Agricultural Products, Shenzhen, China; ^6^ Department of Biology, McMaster University, Hamilton, ON, Canada

**Keywords:** industrial hemp, soil microbiota, rhizosphere microbiome, environmental gradient, community assembly

## Abstract

The southwestern, central, and northeastern regions of China are the primary cultivation areas for industrial hemp. Microorganisms within the soil-root continuum play a crucial role in plant health. However, the mechanisms by which these microbial communities respond to environmental gradients remain unclear. This study aimed to explore how bacterial and fungal communities in the bulk soil and rhizosphere of industrial hemp respond to environmental gradients across diverse climatic zones. We collected soil samples from eight representative regions and analyzed microbial diversity, composition, and assembly mechanisms using DNA metabarcoding. Results showed that microbial diversity in the rhizosphere was lower than in bulk soil, likely due to selective root filtering. The lowest diversity occurred in the temperate continental climate, highlighting the effects of precipitation, soil nutrients, and organic carbon. Climate type was the main factor influencing microbial community structure, with annual precipitation and temperature as key environmental constraints. Bacterial communities were mainly assembled by deterministic processes, while fungal communities were driven by stochastic processes. Additionally, geographic variation in rhizosphere microbial function reflected a co-adaptive mechanism between hemp and its microbial community under varying environmental pressures. These findings enhance our understanding of industrial hemp soil microbiomes and provide insights for optimizing cultivation management.

## 1 Introduction

Industrial hemp, known for its unique economic value, is widely utilized in the oil, textile, and pharmaceutical sectors (Kaur and Kander, 2023; Schumacher et al., 2020). With the rapid expansion of industrial hemp cultivation in China, particularly in the southwestern, central, and northeastern regions, understanding the ecological dynamics of rhizosphere microbial communities is crucial for sustainable hemp production (Ahmed et al., 2021).

The growth and productivity of hemp are intimately influenced by the surrounding soil environment, particularly by the composition and functional attributes of soil microbial communities. These microorganisms enhance plant resilience to abiotic stresses such as drought (Bisht et al., 2025), nutrient deficiency (Li X. H. et al., 2025), and salinity. Beneficial taxa, including species within genera *Bacillus*, *Pseudomonas* and various mycorrhizal fungi, can induce systemic resistance through hormone signaling (e.g., salicylic acid and jasmonic acid), production of osmo-protectants, and stimulation of antioxidant enzyme activity (Lin et al., 2020).

Rhizosphere microbial communities play a pivotal role in promoting plant growth, facilitating nutrient cycling, and enhancing disease resistance (Ling et al., 2022; Marschmann et al., 2024). The rhizosphere effect refers to how plant root exudates and local soil conditions influence microbial composition and activity (López et al., 2023; Lv et al., 2023; Pang et al., 2021). Plant can selectively recruit beneficial microbes by modifying nutrient profiles in the rhizosphere, leading to microbial assemblages distinct from those in bulk soil (Santoyo, 2022; Zhang et al., 2019). For instance, hemp roots have been reported to enrich *Firmicutes* by exuding labile carbon and nitrogen compounds (Li J. et al., 2025).

However, the structure and ecological dynamics of fungal communities in the hemp rhizosphere remain poorly characterized. Studies in other crops have demonstrated that fungal phyla such as *Ascomycota*, *Basidiomycota*, and *Glomeromycota* perform essential functions ranging from organic matter decomposition to enhancing phosphorus mobilization and systemic pathogen resistance. In hemp, rhizospheric fungal community composition may vary with soil type and climatic conditions, potentially impacting plant biomass accumulation and abiotic stress tolerance.

The composition and functional attributes of fungal and bacterial communities can vary substantially between the rhizosphere and bulk soil, shaped by geographic location and edaphic factors (Durán et al., 2022; Li J. J. et al., 2023; Yang et al., 2023). Despite growing interest in plant-associated microbiomes, studies specifically addressing rhizosphere microbial diversity and function in hemp *Cannabis sativa*, particularly within Chinese agroecological systems remain limited. This knowledge gap constrains the development of region-specific cultivation strategies.

Environmental gradients, including geography and climate, play a critical role in shaping microbial community assembly (Debray et al., 2022; Lian et al., 2023). Variations in altitude, mean annual temperature (MAT), and mean annual precipitation (MAP) affect key soil properties such as pH, nutrient availability, and water retention, which in turn influence microbial diversity and structure (Lian et al., 2023). Environmental filtering and dispersal limitation are recognized as key drivers of microbial communities assembly, leading to deterministic or stochastic patterns depending on local habitat conditions (Riddley et al., 2025). Additionally, agricultural practices such as irrigation, pest control and fertilization alter the soil physicochemical properties and can either support or disrupt beneficial microbiome functions (Ablimit et al., 2022).

Despite the recognized ecological significance of soil microbial communities, comprehensive assessments of hemp-associated microbiomes across China’s diverse cultivation regions are still lacking. Therefore, this study aimed to (i) characterize the diversity and structure of bacterial and fungal communities in rhizosphere and bulk soil across eight major hemp-growing regions in China, (ii) assess the influence of environmental and geographic factors on microbial assembly, and (iii) identify functional traits and microbial interactions that contribute to the environmental adaptability of hemp. These findings will provide a theoretical foundation for microbiome-informed strategies to enhance hemp production under regionally variable ecological conditions.

## 2 Materials and methods

### 2.1 Sample collection

In this study, all samples were collected in September 2019 from eight representative industrial hemp cultivation regions across six provinces in China (**Table 1**). At each site, bulk soil and rhizosphere soil samples were collected from healthy plants at the flowering stage. The plants were placed into sterile bags, and the soil that detached with gentle shaking was collected as bulk soil. The samples were transferred to the laboratory on dry ice. The roots were trimmed with sterile scissors and placed into 50 mL sterile centrifuge tubes containing 1x PBS buffer solution (Edwards et al., 2015). The tubes were shaken vigorously to remove soil particles from the root surfaces. After removing the roots with sterile tweezers, the liquid was centrifuged at 5000 rpm for 10 minutes (Zhong et al., 2022). The supernatant was discarded, and the sediment was collected as rhizosphere soil samples. Except for the Lu’an (LA), Anhui rhizosphere samples, which had five replicates due to sample limitations, all other samples were collected in six replicates.

**Table 1:** Information about sampling sites and hemp varieties.

Province	Region	Latitude/Longitude	Varieties	Code	Climatic type
Heilongjiang	Harbin	126.44/45.59	Long *Cannabis* No. 5	HRBA	TC
		126.44/45.59	Long *Cannabis* No. 3	HRBB	TC
	Daqing	125.23/46.67	Qing *Cannabis* No. 1	DQ	TC
Jilin	Changchun	125.09/43.72	Fen *Cannabis* No. 3	CCA	TC
			Qing *Cannabis* No. 1	CCB	TC
Hunan	Yuanjiang	112.36/28.76	Wan *Cannabis* No. 1	YJA	SM
			97 *Cannabis*	YJB	SM
			C-em5 *Cannabis*	YJC	SM
Yunnan	Chuxiong	101.55/25.14	Yun *Cannabis* No. 7	CX	SPM
	Qujing	103.75/25.85	Yun *Cannabis* No. 7	QJ	SPM
Shandong	Tai’an	116.09/35.97	Zhong *Cannabis* No. 5	TA	TC
Anhui	Lu’an	116.52/31.81	Wan *Cannabis* No. 1	LA	SM

### 2.2 Soil physicochemical properties measurement

Available potassium (AK), available nitrogen (AN), available phosphorus (AP), and soil organic matter (SOM) were quantified and analyzed by Wuhan Punas Biotechnology. The climate and physicochemical properties of the soil in the sampling area are shown in **Supplementary Table S1**.

### 2.3 DNA extraction, sequencing, and processing

The total genomic DNA of each soil microbial community was extracted according to the protocol by the E.Z.N.A.^®^ Soil DNA Kit (Omega Bio-tek, Norcross, GA, US). The extracted DNA’s quality and purity were assessed through 1% agarose gel electrophoresis and a NanoDrop 2000 spectrophotometer (Thermo Scintific, US). The V3-V4 region of the 16S rRNA gene of soil bacteria was amplified using universal primers 338F (5’-ACTCCTACGGGAGGCAGCAG-3’) and 806R (5’-GGACTACHVGGGTWTCTAAT-3’) (Kang et al., 2024). The primers for the fungal DNA amplification were ITS1F (5’-CTTGGTCATTTAGAGGAAGTAA-3’) and ITS2R (5’-GCTGCGTTCTTCATCGATGC-3’) (Wang et al., 2019). After the DNA library was constructed, sequencing was carried out on the Illumina MiSeq platform, producing 250 bp paired-end reads. Raw sequences were quality-filtered using Fastp (version 0.14.1) to remove low-quality reads (Q-score < 20). Clean reads were then merged, and chimeric sequences were detected and removed using UPARSE (Edgar, 2013). Operational taxonomic Units (OTUs) were clustered at 97% similarity, and representative sequences were taxonomically assigned using the RDP classifier against the 16S rRNA database (for bacteria) and the UNITE database (for fungi) Sequences affiliated with mitochondria, and OTUs with extremely low abundance (e.g., singletons or < 0.001% of total reads) were filtered out to minimize noise and contamination (Guo et al., 2022). The raw reads were deposited in the NCBI Sequence Read Archive (SRA) database (Bacterial BioProject Number: PRJNA1157812, Fungal BioProject Number: PRJNA1158253).

### 2.4 Data analysis and visualization

Statistical analyses were conducted using the Kruskal-Wallis and Wilcoxon rank-sum tests, with statistical significance adjusted with false discovery rate (FDR). Alpha diversity of different samples was analyzed using Mothur (version v.1.30.2). Beta diversity was assessed by Bray-Curits-based principal coordinate analysis (PCoA) using the package Vegan (v2.4.3) in R (Dixon, 2003). Distance-based redundancy analysis (dbRDA) was performed to examine the associations between microbial communities and environmental variables, with multicollinearity evaluated via vif.cca() (Anderson and Willis, 2003). Variance partitioning analysis (VPA) in the Vegan package was used to quantify the contributions of individual environmental factors to community variation. Random forest regression (R package “randomForest”) was applied to identify key environmental drivers of bacterial and fungal OTU distributions (Breiman et al., 2001). Regression analyses were also conducted between unweighted UniFrac distances and mean annual precipitation (MAP) across soil and rhizosphere communities.

Community assembly processes were further evaluated using βNTI, calculated with the icamp package (v1.5.12). Mantel tests were performed to assess the correlations between community dissimilarity matrices (Bray–Curtis and βNTI) and environmental distance matrices.

Microbial co-occurrence networks were constructed based on OTUs with relative abundances > 0.001 (bacteria) and > 0.01 (fungi), with significant correlations defined as Spearman’s ρ > 0.6 and *p* < 0.01. Network metrics (e.g., nodes, edges, density) were calculated using the ggClusterNet package (Wen et al., 2022). Bacterial ecological functions and metabolic pathways were predicted using FAPROTAX and PICRUSt2, respectively (Sansupa et al., 2021). Fungal functional prediction was performed using FUNGuild (Nguyen et al., 2016), followed by statistical comparison of intergroup differences via the Kruskal-Wallis test.

## 3 Results

To investigate the microbial communities within the soil and rhizosphere of industrial hemp, we selected eight cultivation sites across China, representing a range of environmental gradients. These gradients included climate types (MAP and MAT) and soil nutrients (AN, AP, AK and SOM).

### 3.1 Composition of bulk and rhizosphere microbial communities

**Figure 1:**
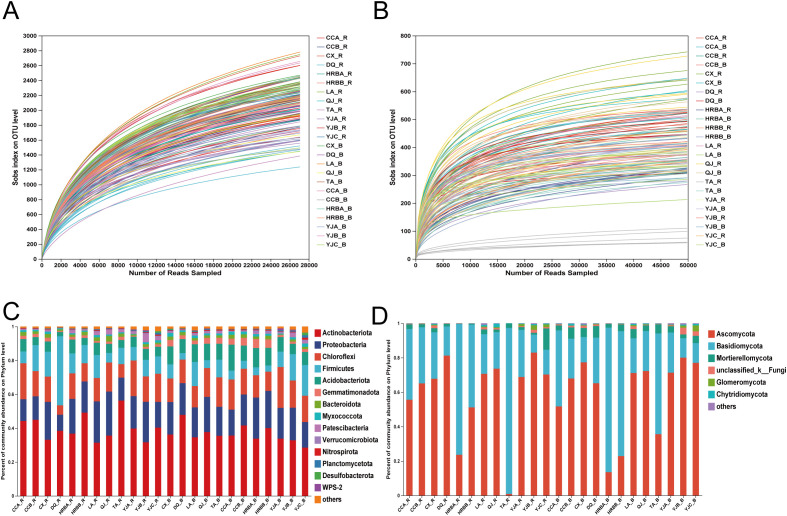
Rarefaction curves and community compositions. **(A, B)** Bacterial/fungal rarefaction curve, respectively. **(C, D)** Relative abundances of bacteria/fungi at phylum level, respectively. B and R, bulk soil and rhizosphere soil, respectively.

Bacterial sequencing generated 7,932,541 paired-end reads across 142 samples, with an average retention of 23,206,457 bp per sample, yielding 6,456 OTUs belonging to 38 phyla, 129 class, 286 order, 448 family, 940 genera, and 2,106 species. Fungal sequencing produced 10,018,879 reads, averaging 16,388,019 bp per sample, resulting in 2,623 OTUs spanning 17 phyla, 55 class, 122 order, 272 family, 577 genera, and 1,038 species. All rarefaction curves showed a clear tendency to reach saturation plateaus, suggesting that the sequencing depth used in this study was adequate to capture the microbial community structure (**Figures 1A, B**).

Overall, the bacterial community composition of hemp in both rhizosphere and bulk soils were similar at the phylum level, mainly composed of *Actinobacteria*, *Proteobacteria Chloroflexi*, *Firmicutes* and *Acidobacteriota* (**Figure 1C**). Similarly, the fungal communities in the rhizosphere and bulk soils of industrial hemp also consisted of taxa from the same three main phyla: *Ascomycota*, *Basidiomycota* and *Mortierellomycota* (**Figure 1D**).

### 3.2 Diversity and structure analysis of soil and rhizosphere microbial communities in industrial hemp

**Figure 2:**
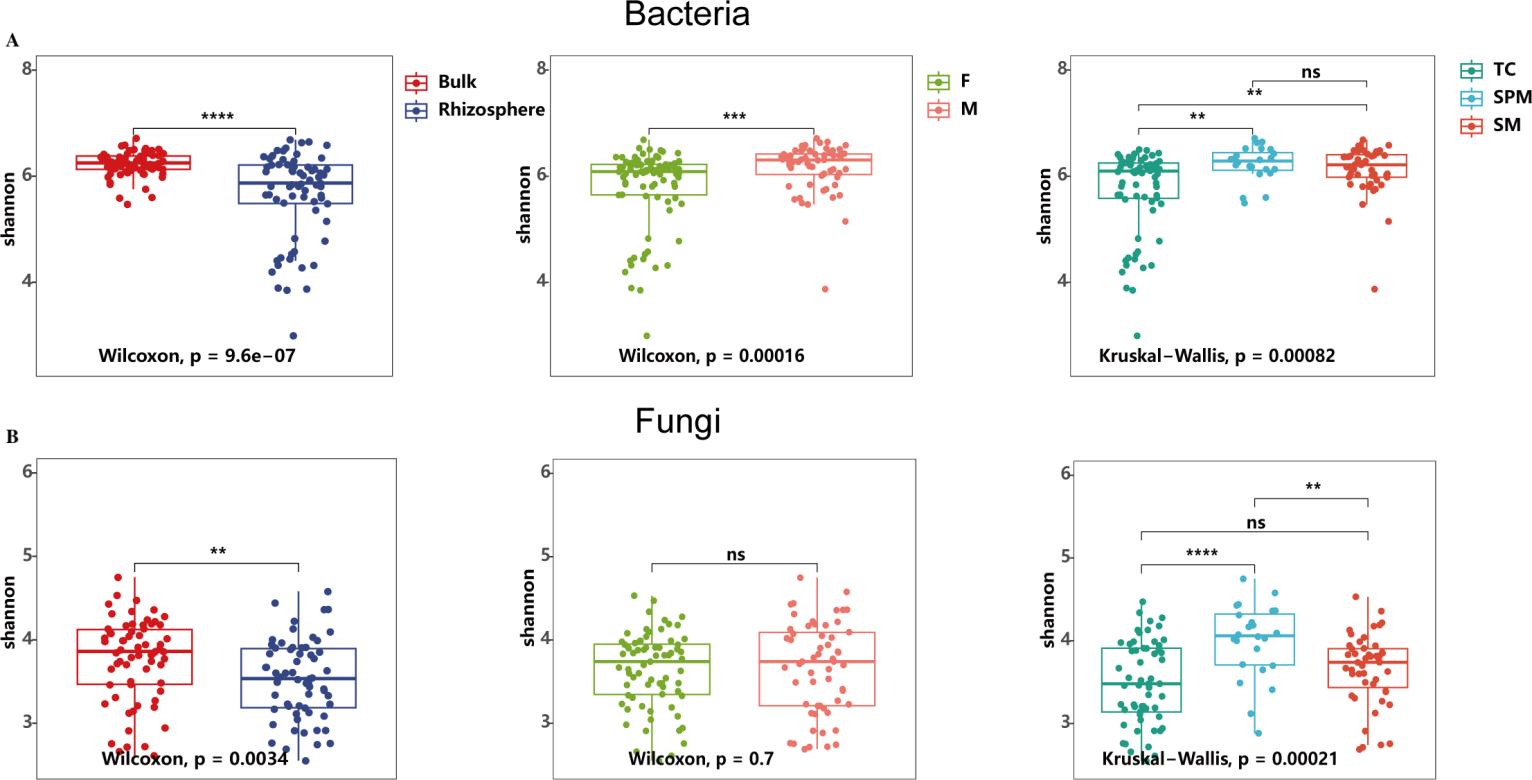
Shannon index-based alpha diversity of bacterial and fungal communities in industrial hemp. **(A)** Bacterial community; **(B)** Fungal community. Rhizosphere, rhizosphere soil; Bulk, bulk soil; F, fiber; M, medicinal; TC, temperate continental climate; SM, subtropical monsoon climate; SPM, subtropical plateau monsoon climate. * p < 0.05; ** p < 0.01; *** p < 0.001; ns, not significant (p ≥ 0.05).

The study revealed substantial differences in the Shannon diversity of bacterial communities across niches, types of use, and climate types. Bacterial diversity in bulk soil was significantly greater than in the rhizosphere (**Figure 2A**, Wilcoxon test, *P <*0.001, **Supplementary Table S2**). Additionally, bacterial diversity in both the bulk and rhizosphere soils of medicinal hemp was significantly higher than that of fiber hemp (**Figure 2A**, Wilcoxon test, *P* < 0.001 and **Supplementary Figure S1**, Wilcoxon test, *P* < 0.05). Climate types also strongly influenced bacterial diversity, with rhizosphere bacterial diversity under the temperate continental (TC) climate being significantly lower than that under the subtropical plateau monsoon (SPM) climate and the subtropical monsoon (SM) climate (**Supplementary Figure S1**, Kruskal−Wallis test, *P* < 0.01).

Similarly, fungal communities exhibited differences in Shannon diversity between rhizosphere and bulk soils (Wilcoxon test, *P* < 0.01), and across climate types (Kruskal−Wallis test, *P* < 0.001). Fungal diversity in the rhizosphere was notably lower compared to bulk soil, with the highest diversity observed under the SPM climate, which was significantly greater than those under SM and TC climates. However, unlike bacterial communities, there was no significant difference in fungal diversity between industrial and medicinal hemp varieties (**Figure 2B**; **Supplementary Figure S1** Wilcoxon test, *P* > 0.05).

**Figure 3:**
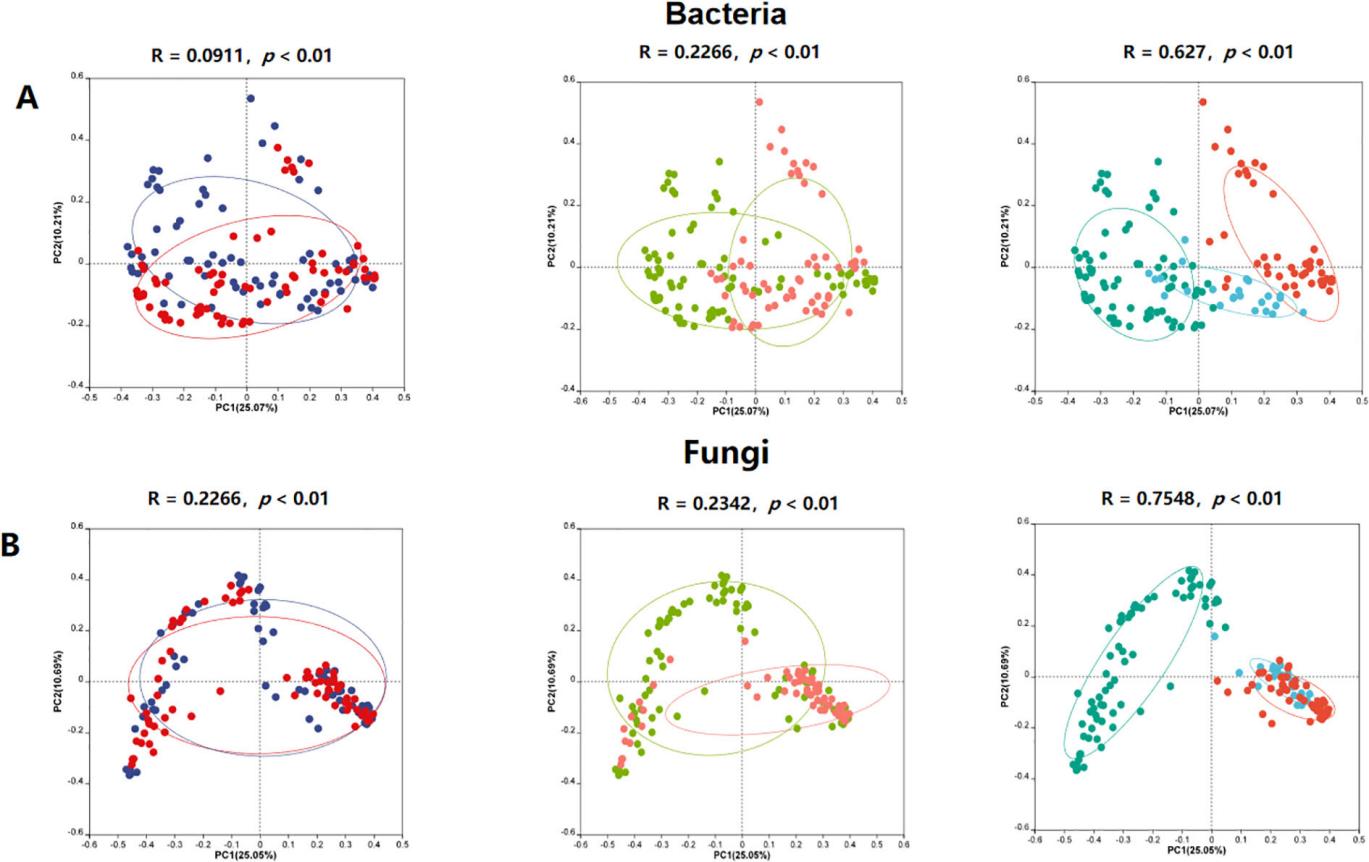
PCoA-based beta diversity of bacterial and fungal communities in industrial hemp. **(A)** Bacterial community; **(B)** Fungal community.

Principal Coordinates Analysis (PCoA) based on Bray-Curtis dissimilarity showed that niches, cultivars, and climate types significantly influenced the structure of both bacterial and fungal communities (*P* < 0.01). Among these factors, climate types had the greatest impact on the structure of bacterial (**Figure 3A**, R_bacteria_ = 0.63) and fungal (**Figure 3B**, R_fungi_ = 0.75) communities. The separation of samples along the PCoA axes highlights the distinct microbial assemblages associated with different environmental conditions.

**Figure 4:**
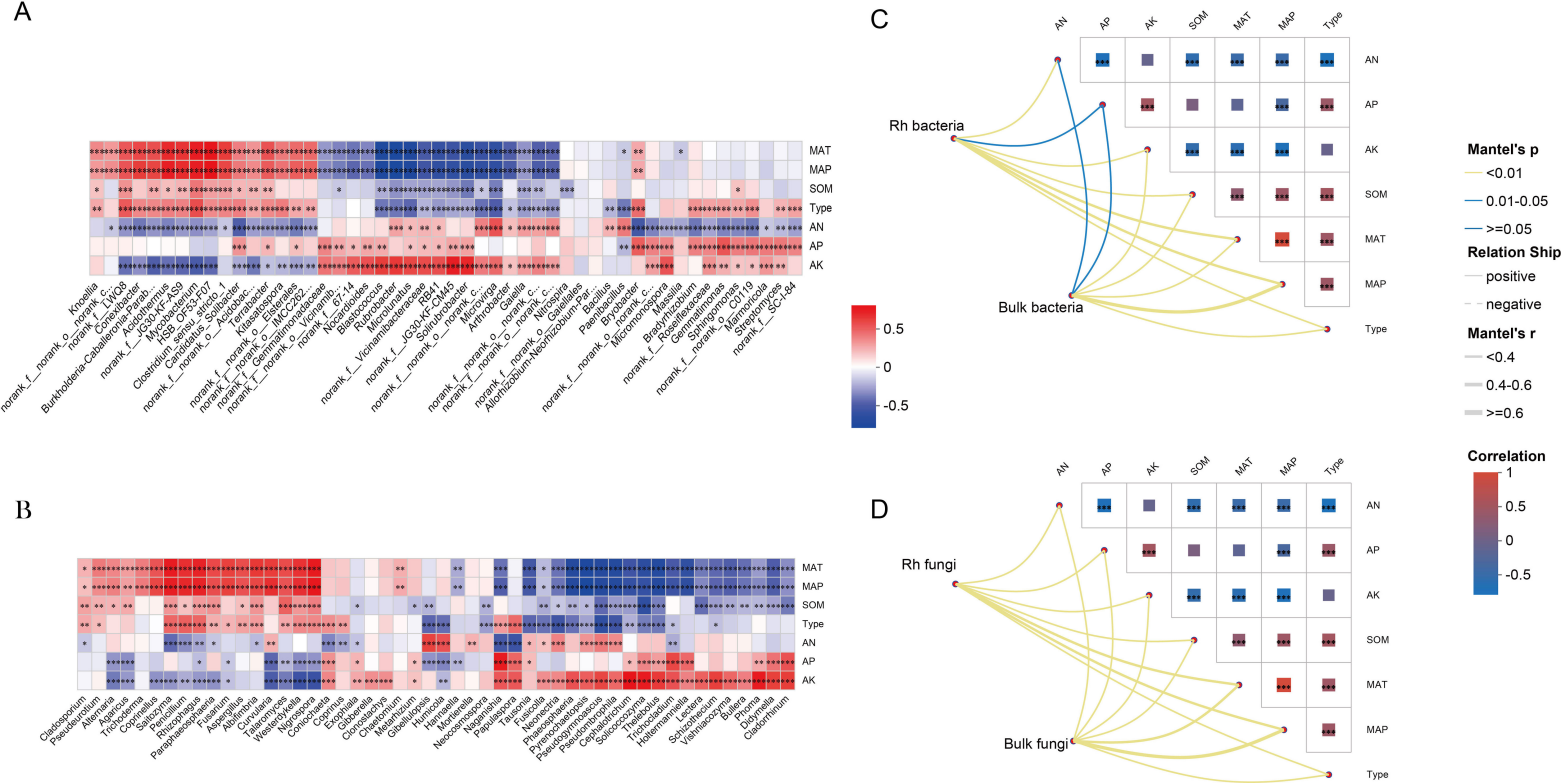
Environmental drivers of community diversity. **(A)** Relationship between bacterial communities and environmental factors. **(B)** Relationship between fungal communities and environmental factors. **(C, D)** Assessing microbial community-environment relationships using the Mantel test. * p < 0.05; ** p < 0.01; *** p < 0.001; ns, not significant (p ≥ 0.05).

Spearman correlation analysis revealed that dominant microbial taxa were significantly influenced by environmental factors, climatic variables, and soil types, all of which notably altered their relative abundance (**Figures 4A, B**). Furthermore, Mantel tests between microbial community structure and environmental variables identified mean annual temperature (MAT) and mean annual precipitation (MAP) as the primary drivers shaping community composition (**Figure 4C**).

### 3.3 Relationship between the microbial community structure and environmental factors in industrial hemp

**Figure 5:**
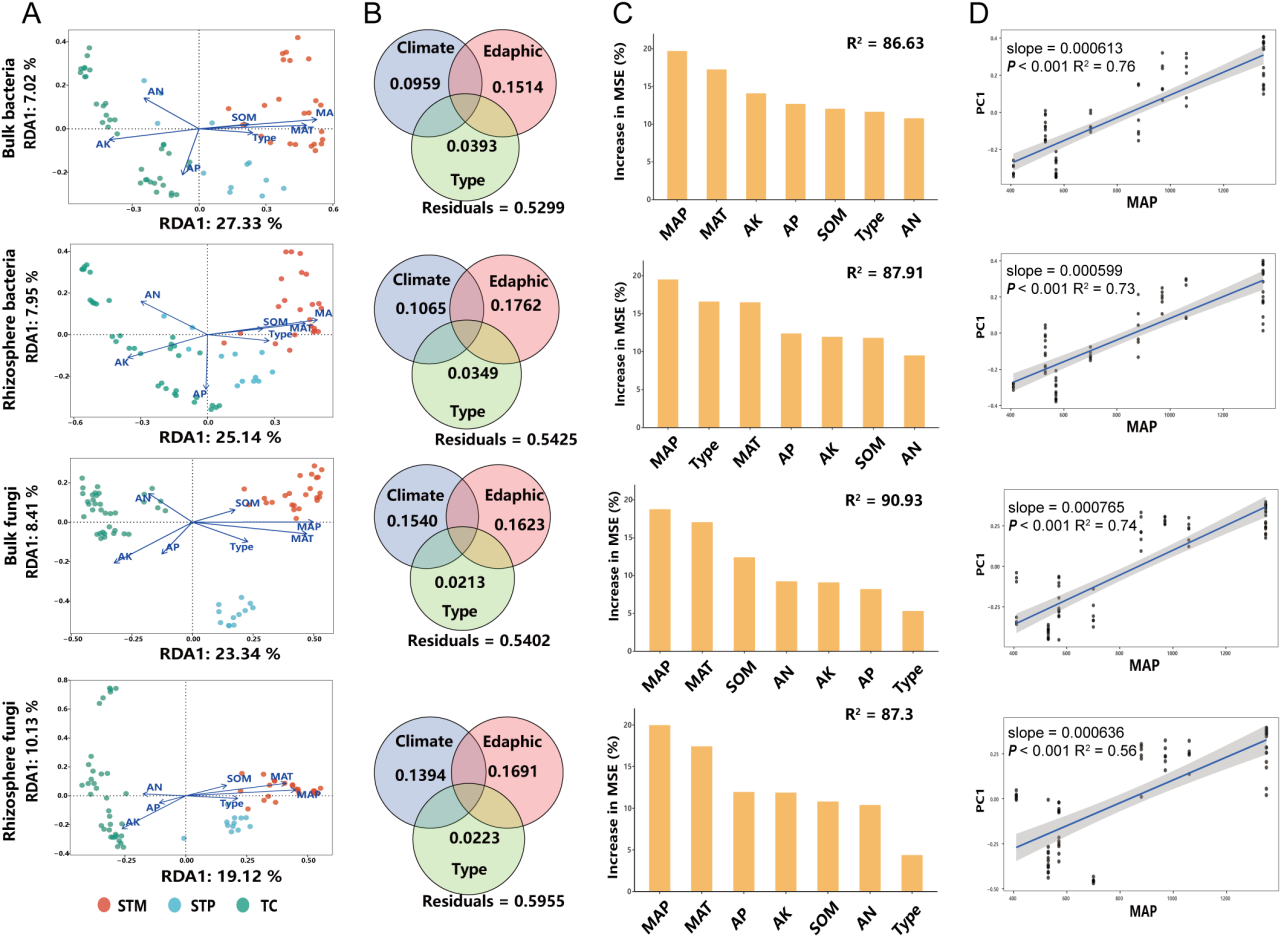
Relationship between microbial communities and environmental factors in soil and rhizosphere of industrial hemp **(A)** Relationships between microbial communities, soil physicochemical properties, and climatic factors. **(B)** VPA results indicating the relative explanatory rates of soil physicochemical properties, climatic factors, and cultivars on bacterial community structure. Eadphic include AN, AP, AK, and SOM, Climatic factors include MAT and MAC; Type include fiber and medicinal types. **(C)** The importance of environmental factors on the composition of rhizosphere microbial communities in industrial hemp. **(D)** The correlation between sample distance and annual mean precipitation.

Redundancy Analysis (RDA) results show that environmental factors explain a significant portion of the variations in microbial community structures in rhizosphere and bulk soil of industrial hemp (**Figure 5A**). For bacterial communities (**Supplementary Table S3**), environmental factors explained 49.87% of the variation in rhizosphere (R² = 0.4987, *P* < 0.001) and 50.45% in bulk soil (R² = 0.5045, *P* < 0.001) samples. Similarly, for fungal communities (**Supplementary Table S3**), 48.93% of the variation in rhizosphere (R² = 0.4893, *P* < 0.001) and 50.73% in bulk soil (R² = 0.5073, *P* < 0.001) samples were due to differences in environmental factors. Among the environmental factors, MAT and MAP had the strongest influence on microbial community structures in both bacterial and fungal communities (**Supplementary Table S3**).

Variance Partitioning Analysis (VPA) further indicated that climate and edaphic factors collectively explained a significant portion of the variation in microbial communities of industrial hemp. Specifically, these factors explained 24.73% (R_Climate_ = 0.0959, R_Edaphic_ = 0.1514) of the variation in bulk soil bacterial communities, 28.87% (R_Climate_ = 0.1065, R_Edaphic_ = 0.1762) in rhizosphere bacterial communities, 31.63% (R_Climate_ = 0.1540, R_Edaphic_ = 0.1623) in bulk soil fungal communities, and 30.85% (R_Climate_ = 0.1394, R_Edaphic_ = 0.1691) in rhizosphere fungal communities (**Figure 5B**). This highlights the importance of climate factors, particularly MAP and MAT, in shaping microbial community dynamics.

Random forest analysis identified MAP as the most important environmental factor influencing soil-rhizosphere microbial communities, followed closely by MAT (**Figure 5C**). Additionally, a significant positive correlation was observed between MAP and the compositional distance of microbial communities in both soil and rhizosphere, suggesting that precipitation plays a critical role in determining microbial diversity and structure (**Figure 5D**).

### 3.4 The assembly mechanisms of soil-rhizosphere microbial communities in industrial hemp

**Figure 6:**
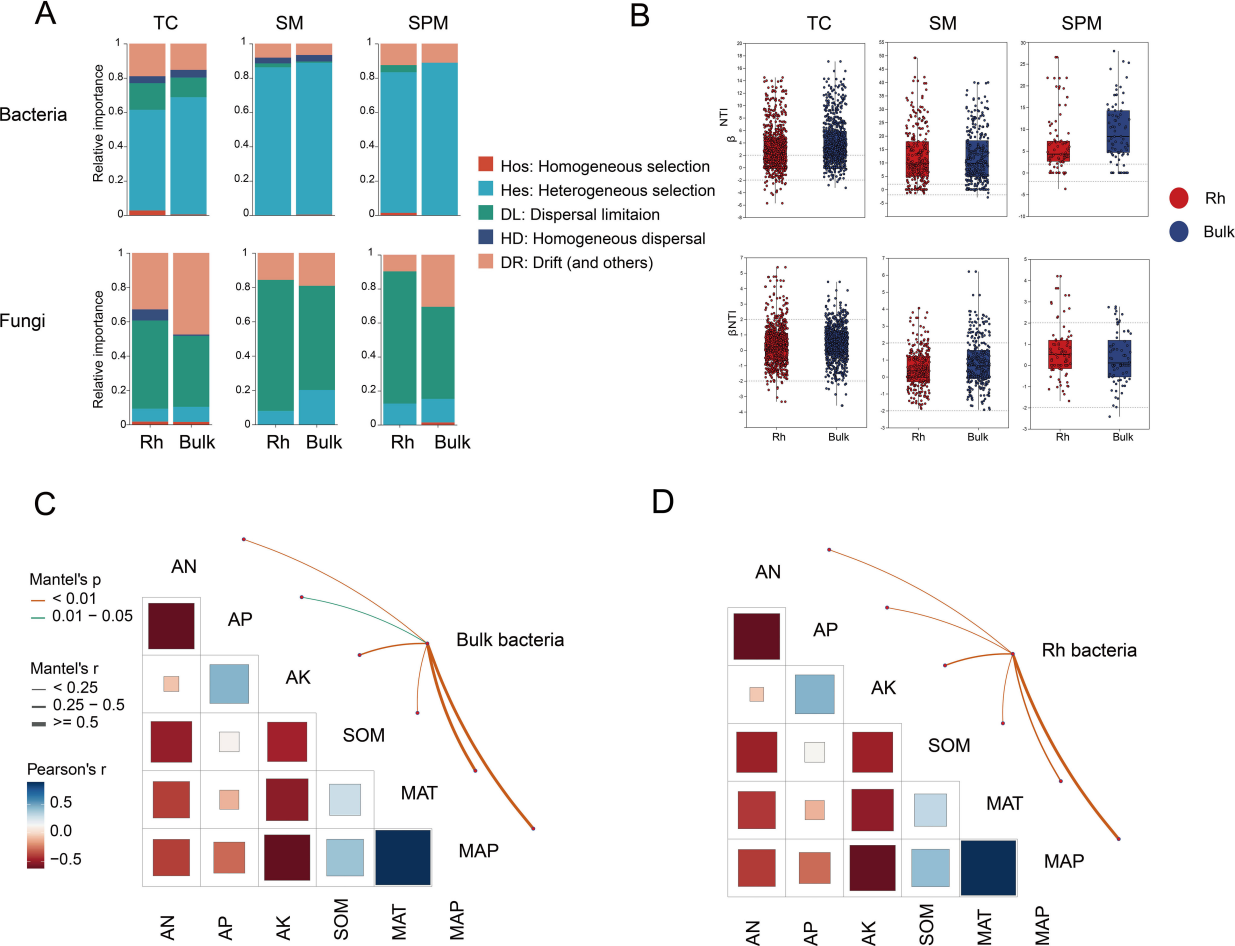
Assembly mechanisms of microbial communities of industrial hemp. **(A)** Assembly of microbial communities. **(B)** βNTI frequency distribution histograms. **(C, D)** Mantel tests correlating environmental factors with |βNTI| values.

The βNTI analysis revealed distinct differences in community assembly mechanisms between bacteria and fungi across rhizosphere and bulk soils. Frequency distribution histograms of βNTI values indicated that bacterial communities were primarily shaped by deterministic processes (|βNTI| > 2), particularly in the SM and SPM regions, where the proportions of deterministic assembly reached 86.2% and 83.34% in rhizosphere soil, and 88.89% in bulk soil for both climates, respectively. In contrast, bacterial communities in the TC region exhibited a comparatively lower proportion of deterministic processes (63.42% in rhizosphere, 68.82% in bulk soil), suggesting weaker environmental filtering under higher climatic stress (**Figures 6A, B**).

Mantel tests between bacterial βNTI matrices and environmental variables further supported these findings, revealing that MAP and MAT were the two most significant factors associated with bacterial community assembly (*P* < 0.01), indicating a strong influence of climate-driven environmental filtering (**Figures 6C, D**).

In contrast, fungal community assembly was largely governed by stochastic processes (|βNTI| < 2), as indicated by the low proportion of deterministic influence (**Figure 6A**). In rhizosphere, deterministic processes accounted for only 7.94%, 12.5%, and 10.34% of assembly in the SM, SPM, and TC regions, respectively. Similarly, in bulk soils, the proportions remained low, at 20.14% (SM), 15.28% (SPM), and 10.34% (TC) (**Figure 6B**). These findings highlight a strong dispersal limitation in fungal communities, especially under varying environmental conditions, and reinforce the notion that while bacterial communities tend to respond more directly to environmental gradients, fungal communities are assembled more randomly, potentially due to differences in ecological traits and dispersal strategies.

### 3.5 Co-occurrence networks of soil-rhizosphere microbial communities of industrial hemp under environmental factors

**Figure 7:**
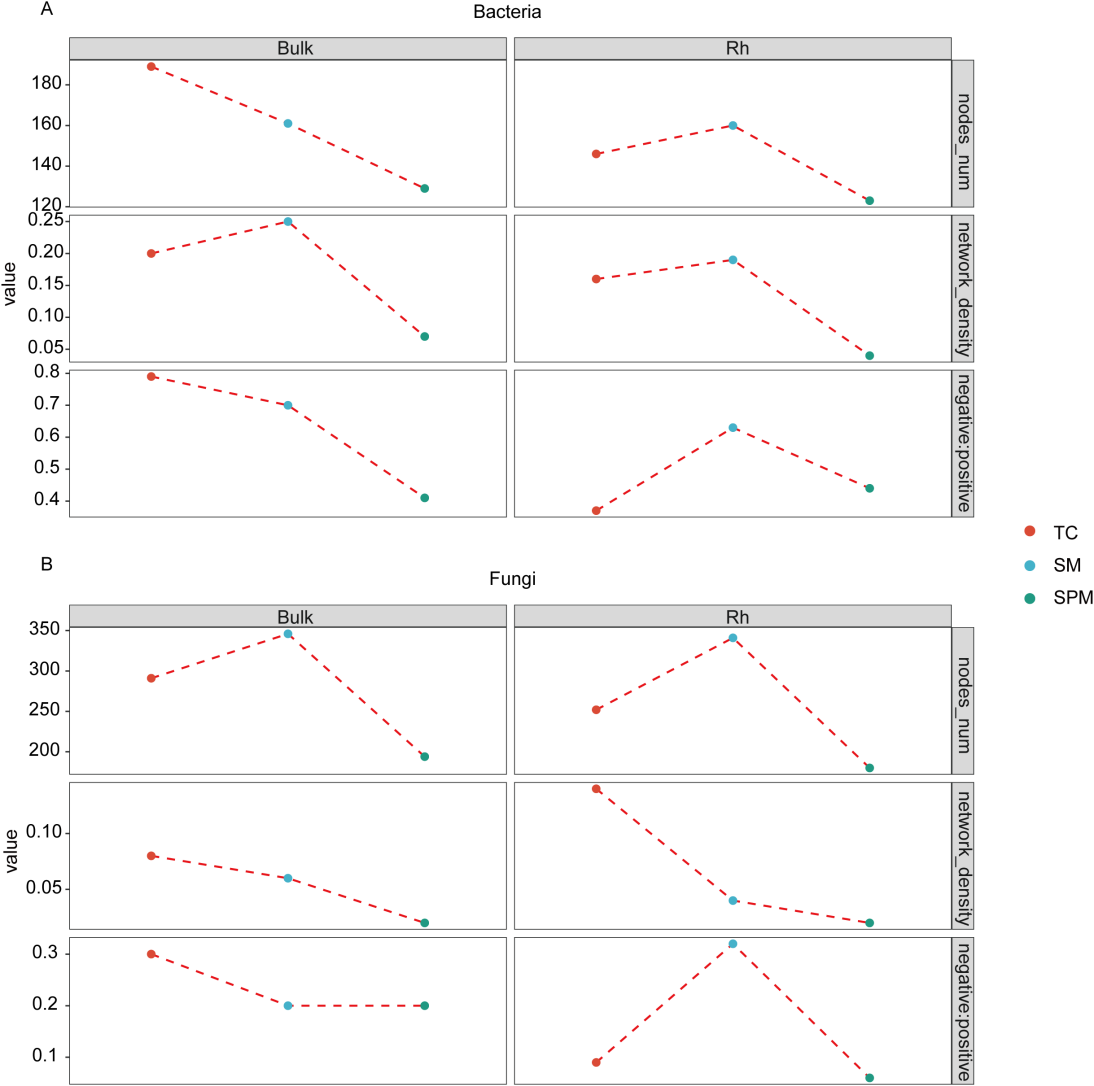
Co-occurrence networks of microbial communities in the soil-rhizosphere of industrial hemp under environmental conditions. **(A)** Bacterial community; **(B)** Fungal community.

Co-occurrence network analysis revealed distinct alterations in microbial topological features along the environmental gradient (**Figure 7**). The total number of nodes in both bulk soil and rhizosphere microbial networks was generally lower in the TC and SPM climatic zones compared to SM, with the exception of the bacterial network in TC bulk soil. Notably, the rhizosphere bacterial network in SM exhibited the highest nodes (160, **Figure 7A**), while the corresponding fungal network comprised 341 nodes (**Figure 7B**). In contrast, the SPM rhizosphere networks showed the lowest complexity, with only 123 bacterial and 180 fungal nodes (**Figures 7A, B**).

Network density also differed across climate types. Bacterial networks in SM demonstrated higher density relative to those in TC and SPM, whereas fungal networks exhibited the highest density under TC. Furthermore, the balance of microbial interactions showed clear habitat-specific patterns. The ratio of negative-to-positive correlations in rhizosphere bacterial networks was lowest in TC (0.37), suggesting more cooperative interactions, and increased to 0.73 and 0.41 in SM and SPM, respectively. A similar trend was observed in rhizosphere fungal networks, indicating consistent shifts in microbial interaction dynamics along the climate gradient.

### 3.6 Functional prediction of soil-rhizosphere bacteria in industrial hemp across climate types

**Figure 8:**
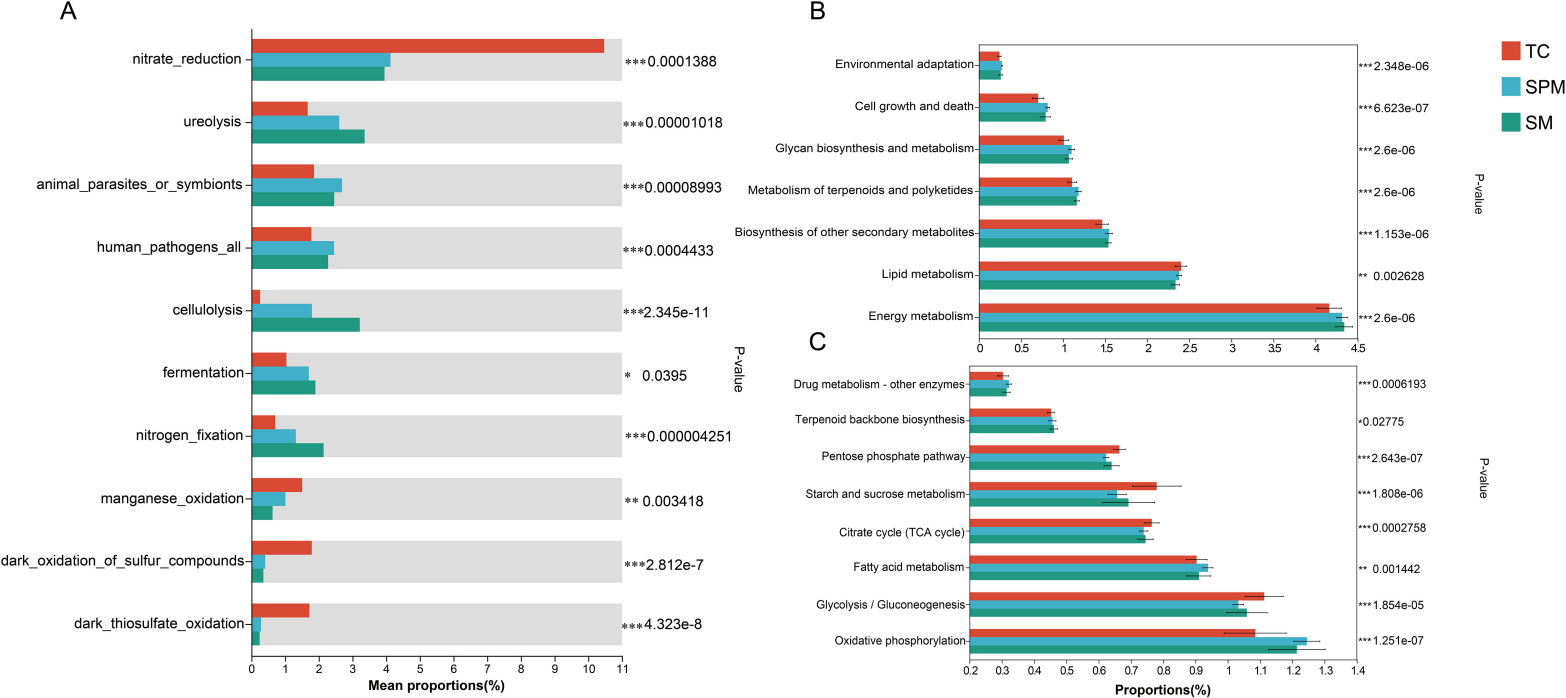
Variation in rhizosphere functional bacteria of industrial hemp among cultivation regions. **(A)** Climate-driven variations in bacterial functional profiles predicted by FARAPTOX. **(B)** Climate-driven variations in bacterial functional profiles at KEGG level 2. **(C)** Climate-driven variations in bacterial functional profiles at KEGG level 3.

Functional annotation based on the FAPROTAX revealed significant differences in several ecological functions among climatic regions (**Figure 8A**). Notably, nitrate_reduction was significantly enriched in TC (*P* < 0.001), whereas ureolysis and cellulolysis were significantly depleted in TC compared to the other groups (*P* < 0.001). Fermentation (*P* < 0.05) and nitrogen fixation (*P* < 0.001) also exhibited significant regional variation. In addition, manganese oxidation and dark thiosulfate oxidation were predominantly enriched in the TC.

Further functional prediction using PICRUSt2 identified significant differences (**Figure 8B**) in several level-3 KEGG metabolic pathways among groups (**Figure 8C**). Drug metabolism pathways were substantially enriched in SPM (*P* < 0.001). Significant variation was also observed in the citrate cycle and fatty acid degradation across all groups. Oxidative phosphorylation was notably enriched in SPM and SM (*P* < 0.001), while glycolysis/gluconeogenesis exhibited only minor variation, with slightly elevated levels in TC.

**Figure 9:**
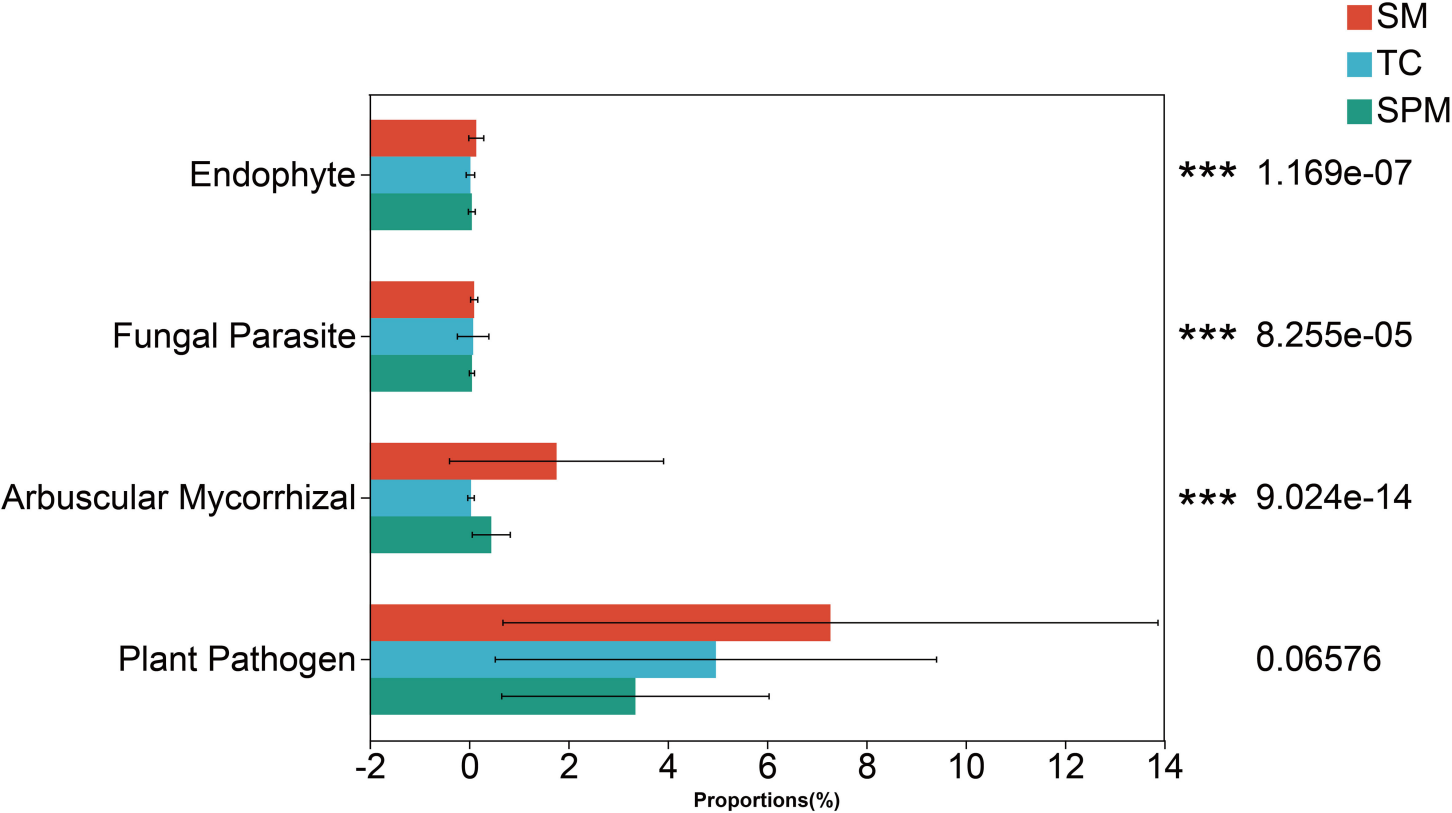
Variation in rhizosphere functional fungi of industrial hemp among cultivation regions.

Functional predictions based on fungal community composition indicated substantial differences in the relative abundance of Endophyte, Fungal Parasite and Arbuscular Mycorrhizal fungi across industrial hemp rhizosphere communities from different climatic regions (**Figure 9**).

## 4 Discussion

In this study, we investigated the diversity, composition, assembly of microbial communities and co-occurrence networks of microbial communities in the soil-rhizosphere continuum of industrial hemp across a broad climate gradient from northeast to southwest China. The study found that: (i) Climate types significantly influence the diversity of rhizosphere-associated microbial communities in industrial hemp, with MAP being the most important influencing factor. (ii). Deterministic processes, particularly heterogeneous selection, play an important role in the assembly of rhizosphere and soil bacterial communities in industrial hemp, while stochastic processes, particularly dispersal limitation, are key in the assembly of rhizosphere and soil fungal communities in industrial hemp. (iii). Environmental stress amplifies the distinctions between microbial communities in the rhizosphere and soil.

### 4.1 Influence of niche and environmental conditions on microbial communities in soil and rhizosphere of industrial hemp across environmental gradients

The variations in microbial community diversity and composition within the soil-rhizosphere continuum of industrial hemp were significantly influenced by niche, as shown in both bacterial and fungal communities (**Figures 1**, **2**, **3**). Specifically, rhizosphere microbial diversity was significantly lower than that in bulk soil, likely due to the selective filtering effect of root, which shape microbial compositions by recruiting or excluding certain taxa from the external environment (Maciá-Vicente et al., 2020). This pattern has been observed in other crops such as rice (Iniesta-Pallarés et al., 2023), *Arabidopsis* (Bai et al., 2015), and maize (Peiffer et al., 2013), suggesting a common mechanism across plant species.

In regions characterized by the TC climate type, both bacterial and fungal diversities were the lowest, indicating that environmental stressors such as lower precipitation and nutrient availability may enhance the root’s selective filtering effect (Holz et al., 2024). Our results revealed that climate factors (**Figure 4**), particularly MAP, soil nutrients and SOC, played significant roles in shaping microbial community structure, consistent with previous studies (Junkins et al., 2022; Wang et al., 2022). Climate types explained the largest proportion of variation in both bacterial and fungal communities, showing that the pivotal role of climate in driving microbial composition and function in the soil and rhizosphere.

While plant species have been shown to influence microbial community composition (Li Y. et al., 2023), our findings suggest that varietal differences in industrial hemp only significantly affect bacterial diversity, with no notable impact on fungal diversity. From SM to TC climates, both MAP and MAT showed a decreasing trend, and corresponding changes in soil nutrients indicated that the TC climate is under higher environmental stress. Interestingly, although soil organic matter is typically associated with microbial composition, our random forest analysis highlighted MAP as the most critical environmental constraint, followed by MAT. Additionally, we observed that fungal communities were more sensitive to climate factors than bacterial ones, suggesting that soil and rhizosphere fungi in industrial hemp are more vulnerable to changes in climate conditions, particularly precipitation and temperature.

Finally, the relative abundance of functional bacterial communities showed significant differences across climate types (**Figure 6**), indicating that under different environmental stress conditions, plants recruit specific beneficial microorganisms to promote growth and alleviate stress.

### 4.2 Environmental factors influence the selection and assembly of microbial taxa in the soil and rhizosphere

The βNTI model results reveal distinct construction mechanisms for microbial communities in rhizosphere and non-rhizosphere soils of industrial hemp. Bacterial communities in both environments are predominantly assembled through deterministic processes, with homogeneous selection being the most prominent, while heterogeneous selection plays a minimal role. In contrast, fungal communities are primarily governed by stochastic processes (**Figure 5**). This divergence suggests that while bacterial communities are shaped by uniform environmental conditions, fungal communities are more susceptible to random events. Below we briefly discuss the effects of specific environmental factors on microbial community assembly.

Influence of Root Exudates: Industrial hemp root exudates, rich in organic compounds like carbohydrates, amino acids, and organic acids, provide abundant resources for bacteria, promoting their proliferation and leading to homogenized bacterial communities (Chari and Taylor, 2022). Fungi, however, may utilize these exudates differently, making their community composition more influenced by stochastic processes. Environmental Filtering: Stable soil physicochemical properties (e.g., pH and nutrient content) in hemp cultivation environments strongly select for homogeneous bacterial communities. In contrast, fungi may respond more variably to these conditions, leading to a greater influence of stochastic processes in their assembly (He et al., 2023; Medriano et al., 2023). Agricultural Management Practices: Uniform practices such as fertilization, irrigation, and pest control standardize soil conditions, promoting homogeneous selection for bacterial communities. Fungal communities, however, may exhibit more variable responses to these practices, resulting in a stronger role for stochastic processes (Schmidt et al., 2019). Microbial Interactions: Bacteria may more readily form stable, homogeneous communities through competitive and cooperative interactions (Niehaus et al., 2019), while fungal interactions are more diverse and complex, making their assembly more stochastic in nature (Pierce et al., 2021). Together, these findings highlight the importance of considering the differing assembly mechanisms of microbial communities when studying and managing agricultural ecosystems. Recognizing these differences is crucial for optimizing microbial functions and services in such environments.

### 4.3 Co-occurrence networks of microbial communities in the soil-rhizosphere of industrial hemp across different climate types

Extensive research has demonstrated that ecological networks with a higher ratio of negative to positive correlations among microbial communities are more stable in response to environmental changes, as negative interactions can mitigate disturbances within the community (Zhong et al., 2022). Additionally, under higher environmental stress, positive correlations among community members tend to appear more frequently. Our study found that in the bulk microbial communities of industrial hemp (**Figure 7**), the ratio of negative to positive correlations gradually decreased from TC to SPM, accompanied by an overall decline in network density. This observation is consistent with previous studies and further supports the role of negative correlations in enhancing community stability.

In contrast, the rhizosphere microbial communities exhibited a different pattern, where the ratio of negative to positive correlations first increased and then decreased. This phenomenon may be influenced by the rhizosphere effect, whereby plant roots release exudates that alter the structure and function of the microbial community, resulting in distinct interaction patterns across different climate types.

### 4.4 Functional adaptations of the microbiome under climatic gradients

Functional predictions derived from FAPROTAX, FUNGuild, and KEGG collectively revealed geographically structured microbial traits related to nutrient metabolism, stress adaptation, and disease suppression. This convergence across independent annotation platforms underscores a robust ecological signal, where industrial hemp harbors a functionally adapted microbiome that mirrors environmental constraints.

In nitrogen-limited regions, the enrichment of nitrogen and ureolysis functions-primarily contributed by genera such as Rhizobium, suggests a compensatory recruitment strategy by the plant host to optimize nitrogen acquisition. This observation aligns with the concept of host-mediated microbial selection, where plant exudates and immune signaling modulate microbial assembly toward functional niches that satisfy nutritional demands (Pang et al., 2021). Notably, such processes may not require high taxonomic specificity but instead leverage functional redundancy, whereby multiple taxa perform similar ecological roles, allowing resilient adaptation under variable nutrient regimes.

KEGG inference further revealed enhanced abundance of transport systems, signal transduction pathways, and secondary metabolite biosynthesis genes (e.g., antibiotics, glutathione, oxidative stress response) in rhizosphere communities from arid or climatically variable regions. These functional signatures likely reflect adaptive strategies of both plants and microbes to cope with abiotic stress and inter-organismal competition. For instance, the elevated representation of ABC transporters and two-component systems may facilitate efficient nutrient uptake and root–microbiome communication, while stress-associated pathways like oxidative phosphorylation and glutathione metabolism indicate microbial contributions to detoxification and oxidative stress buffering, functions known to indirectly support plant stress resilience.

These findings support the hypothesis that deterministic environmental filtering, particularly by factors like precipitation and soil nutrients, can drive convergent functional adaptation across microbial communities, even when taxonomic composition remains stochastic. This eco-functional consistency underscores the emergence of a core functional microbiome in hemp cultivation systems. Such microbial assemblages, shaped by environmental gradients yet maintaining key plant-beneficial traits, highlight the adaptive plasticity of the rhizosphere and its critical role in sustaining plant health under changing agroecological conditions.

## 5 Conclusion

The diversity and composition of microbial communities in the soil-rhizosphere continuum of industrial hemp are significantly influenced by niche and environmental factors. Rhizosphere bacterial and fungal communities exhibit lower diversity than bulk soil due to selective root filtering. The lowest diversity was observed in the TC climate type, where the ratio of negative to positive correlations in the microbial community network is also the lowest, highlighting the impact of precipitation, soil nutrients, and soil organic carbon. Climate types accounted for the largest variation in microbial community structures, with mean annual precipitation and mean annual temperature emerging as critical environmental constraints. Bacterial community assembly is predominantly governed by deterministic processes with homogeneous selection, influenced by root exudates, stable soil properties, uniform agricultural practices, and microbial interactions. In contrast, fungal communities are driven by stochastic processes due to varied utilization of root exudates, diverse responses to environmental conditions, less uniform responses to agricultural practices, and complex microbial interactions. The geographic variation in rhizosphere microbial functions reflects the co-adaptive mechanisms between industrial hemp and its microbial community under different environmental pressures. These findings underscore the need to consider these differences in microbial community assembly for optimizing agricultural ecosystem management.

## Data Availability

The datasets presented in this study can be found in online repositories. The names of the repository/repositories and accession number(s) can be found below: https://www.ncbi.nlm.nih.gov/, PRJNA1157812 https://www.ncbi.nlm.nih.gov/, PRJNA1158253.

## Supplementary material

Supplementary Figure 1 Shannon index-based alpha diversity of microbiome communities in industrial hemp.
