# Supplementary figures and images for: Functional differentiation of industrial hemp rhizosphere microbiome along environmental gradients
Source: Front Plant Sci. 2025 Aug 20;16:1578662. doi: 10.3389/fpls.2025.1578662 (PMC12405162; doi:10.3389/fpls.2025.1578662)

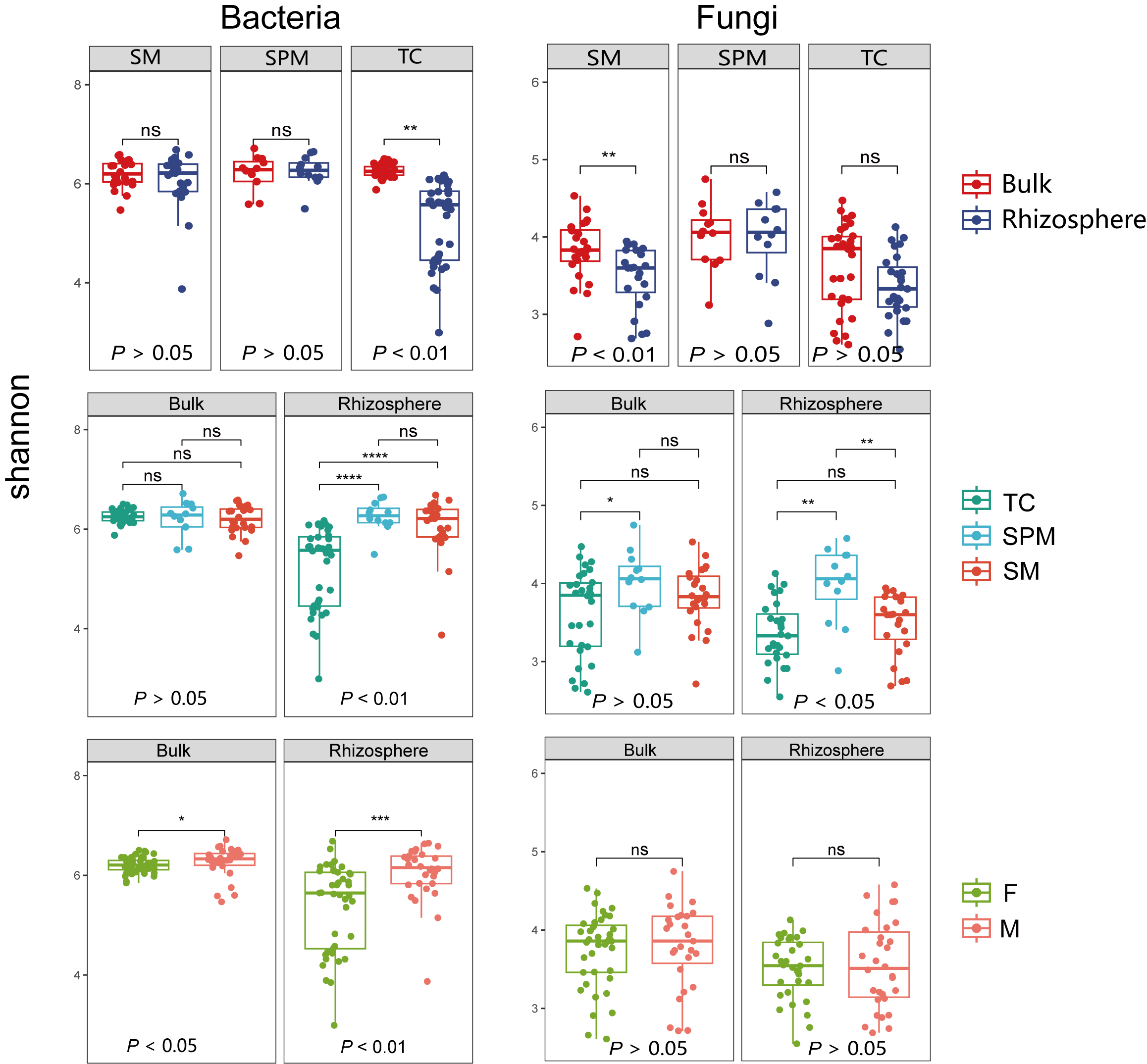

Supplement: Supplementary Figure 1 — Shannon index-based alpha diversity of microbiome communities in industrial hemp. [file Image1.tif]
